# MAGE-A8 overexpression in transitional cell carcinoma of the bladder: identification of two tumour-associated antigen peptides

**DOI:** 10.1038/sj.bjc.6601968

**Published:** 2004-06-22

**Authors:** E Bar-Haim, A Paz, A Machlenkin, D Hazzan, B Tirosh, L Carmon, B Brenner, E Vadai, O Mor, A Stein, F A Lemonnier, E Tzehoval, L Eisenbach

**Affiliations:** 1 Department of Immunology, Weizmann Institute of Science, POB 26, Rehovot 76100, Israel; 2 Department of Urology, Barzilai Medical Center, Ashkelon, Israel; 3 Department of Surgery B, Carmel Medical Center, Haifa, Israel; 4 Institute of Oncology, Rabin Medical Center, Beilinson Campus, Petah Tiqva, Israel; 5 QBI Enterprises Ltd, Weizmann Scientific Park, Ness Ziona, Israel; 6 Department of Urology, Carmel Hospital, Haifa, Israel; 7 AIDS – Retrovirus Department, Antiviral Cellular Immunity Unit, Pasteur Institute, Paris, France

**Keywords:** TAA, MAGE, TCC, CTL, immunotherapy

## Abstract

Bladder carcinoma is the fourth most common cancer in men and the eighth most common cancer among women. Our study is aimed to characterise tumour-associated antigen peptides of transitional cell carcinoma of the bladder (TCC). A DNA micro-array-based differential display analysis of 10 000 genes was carried out, and MAGE-A8 gene expression was detected in the tumour, and not in the normal bladder. High occurrence of MAGE-A8 expression was observed in fresh tumour samples (17 out of 23) and TCC lines (four of eight). The MAGE-A8 protein sequence was screened for HLA-A2.1-binding motifs, six potential peptides were synthesised, and peptides binding to HLA-A2.1 were assured. Immunogenicity and antigenicity of the MAGE-A8 peptides were examined in the HHD system, murine class I MHC knockout mice, transgenic for HLA-A2.1. The MAGE-A8 peptide immunogenicity was examined in three modes of vaccination, delivered intranasally with cholera toxin, injected into the tail base with complete Freund's adjuvant (CFA), or presented directly as loaded onto cell surface HLA-A2.1 molecules. Two peptides, 8.1 and 8.3, induce CTL that kills the T24 TCC line *in vitro*, and prime human lymphocyte response of healthy donors. These results demonstrate the potential use of the MAGE-A8 peptides for specific immunotherapy of TCC.

Bladder cancer is the fifth common cancer in the USA, 95% of the bladder cancers are defined as transitional cell carcinoma (TCC) (Patton *et al*, 2002). There were 263 000 cases of bladder cancer diagnosed in the world, its incidence per 100 000 people is 9.9 in men and 2.3 in women.

Nonspecific immunotherapy with *Bacillus Calmette-Guerin* (BCG), delivered intravesically, is the most active agent in the treatment of superficial bladder cancer in reducing recurrence and progression rate (Nseyo and Lamm, 1997). That may imply the potential benefit of immunotherapeutic treatment for bladder carcinoma.

Tumour-associated antigen (TAA) peptides are 8–10 amino acids long, presented by class I MHC to cytotoxic, CD8^+^ T cells of the immune system (Renkvist *et al*, 2001). Among TAA groups are the differentiation antigens, such as Tyrosinase, gp100 and MART1/Melan-A in melanoma, and PSA in prostate carcinoma. Tumour-associated antigens also originate from overexpressed genes of the tumour, such as HER2/neu, CEA, or uniquely expressed genes of the tumour, such as the MAGE gene family. There are cases of viral proteins that serve as TAAs, such as human papilloma virus 16 (HPV16)-E6, HPV-E7, and HTLV, or Hepatitis B and C antigens, and mutated genes, as in the case of *β*-catenin, CDK4, and CASP8. A case of fusion protein as a source for TAA is of the *bcr-abl* of CML (reviewed in Renkvist *et al*, 2001).

The MAGE family was originally identified as genes that are strictly expressed in tumour cells, but silent in normal adult tissues, except in the male germ line and for some of the genes in the placenta (Chomez *et al*, 2001). The first member of the MAGE family was identified as a gene encoding for tumour-specific antigen (van der Bruggen *et al*, 1991). A cluster of 12 MAGE genes was later recognised, designated MAGE-A 1–12, it was mapped to the q28 arm of the X chromosome (De Plaen *et al*, 1994). Two more clusters were later identified, MAGE-B and MAGE-C, all the genes in the three subfamilies are characterised by a long-terminal exon, encoding the entire protein, and their expression is restricted to tumours. Other subfamilies, MAGE-D, (Lucas *et al*, 1999) and MAGE-E–L (Chomez *et al*, 2001) were also identified. Some of the MAGE proteins take part in cell cycle regulation (Barker and Salehi, 2002).

MAGE-A8 expression in normal tissues is limited to the testis and the placenta (De Plaen *et al*, 1994). Its expression was examined in 80 pairs of normal colon and colorectal carcinomas, the MAGE-A8 gene was expressed in 44% of the tumour samples, and not expressed in any of the 80 normal tissue (Hasegawa *et al*, 1998). An expression frequency of 46% was observed in 22 samples of hepatocellular carcinoma (Tahara *et al*, 1999).

Among MAGE proteins, the expression of MAGE-A1–4 was examined in 57 samples of primary TCC, and varied from 21% (MAGE-A1) to 35% (MAGE-A2), with higher expression level in advanced tumours (Patard *et al*, 1995). An HLA-Cw7 restricted antigen, encoded by the MAGE-A12 gene, was identified using a bladder carcinoma line and an autologous CTL clone specific for the tumour (Heidecker *et al*, 2000). Another bladder carcinoma antigen was identified by autologous CTL, restricted to HLA-B4403. The antigen is originated from a point mutation in a ubiquitously expressed protein designated KIAA0205. The point mutation was not identified in more than 100 tumours of various histologic types (Gueguen *et al*, 1998).

The HHD system was developed in order to screen for HLA-A2.1 restricted antigens in a stable and reproducible murine system. HHD mice, a combination of classical HLA transgenesis and selective destruction of murine H-2, are D^b^ × *β*2-microglobulin (*β*2M) null mice, transgenic for a modified HLA-A2.1-*β*2-microglobulin single chain (Pascolo *et al*, 1997). It was demonstrated that HHD mice selected the same immunodominant CTL epitopes recognised by PBL in influenza-infected HLA-A2.1 individuals, and, unlike traditional HLA transgenic mice, there is no murine background response (Pascolo *et al*, 1997). Hence, these mice are a useful tool for the identification and characterisation of potential tumour-derived, HLA-A2.1 restricted, CTL epitopes. HLA-A2.1 is the most widespread allele of HLA in the human population, therefore our allele of choice for the search of tumour CTL epitopes. The HHD system enabled the identification of TAA of breast carcinoma (Carmon *et al*, 2000, 2002), and colon carcinoma (Tirosh *et al*, 2003). A MAGE-A1 epitope of HLA-A2.1 was recently identified (Pascolo *et al*, 2001), as well as shared epitopes of MAGE genes (Graff-Dubois *et al*, 2002).

The current study aims at identification of HLA-A2.1-restricted CLT epitopes of human bladder carcinoma. A DNA-chip-based differential display analysis was performed, to identify overexpressed and uniquely expressed genes of TCC, as compared to the normal bladder. The MAGE-A8 gene was overexpressed in the tumour, and not expressed in the normal tissue. A high-expression level of MAGE-A8 was also demonstrated in TCC samples. MAGE-A8 CTL epitopes were defined in this study.

## MATERIALS AND METHODS

### Mice

The derivation of HLA-A2.1/D^b^-*β*2 monochain, transgenic, H-2D^b^ × *β*2 m double-knockout mice (HHD mice) has been described by Pascolo *et al* (1997). Mice were bred in the Weizmann Institute of Science (Rehovot, Israel) and maintained and treated according to NIH guidelines.

### Cells

The following human TCC lines were utilised in this study: RT4, T24, J82, TCCSUP, UM-UC3, SW780, 5637, and 1196. T24 is HLA-A2 negative, it was transfected with the HHD construct to give the T24 HHD clone. The TCC lines were maintained in McCOY's 5A medium, containing 10% FCS, 1 mM glutamine, combined antibiotics, 1 mM sodium pyruvate, and 1% nonessential amino acids. T24-HHD transfectants were maintained in the same medium, added with 500 *μ*g ml^−1^ of Geneticin (Life Technologies Inc, Paisley, UK).

RMA-S is a TAP-2-deficient lymphoma clone of C57BL/6 origin. The RMA-S/HHD clone is an HHD transfectant of RMA-S cells. The RMA-S/HHD/B7.1 clone is an HHD transfectant expressing the murine B7.1 co-stimulatory molecule. EL4-HHD is a murine thymoma, transfected with the HHD construct. T2 is a TAP-2-deficient lymphoblastoid line of HLA-A2.1 genotype.

RMA-S and T2 cells were maintained in RPMI-1640 containing 10% FCS and combined antibiotics. RMA-S/HHD/B7 and EL4-HHD cells were maintained in the same medium, added with 500 *μ*g ml^−1^ of Geneticin (Life Technologies Inc, Paisley, UK).

### DNA chip-based differential display

Tissue samples from 10 TCC tumours and 10 transitional cell layers of normal bladder samples were collected during surgery and immediately frozen in liquid nitrogen (Hasharon Hospital, Petach Tikva, Israel). RNA was extracted from each sample separately using EasyRNA kit (Beit Haemek, Israel) and checked for its integrity by agarose gel analysis. In all, 5 *μ*g of each of the 10 TCC samples was pooled. The pooled 50 *μ*g of TCC and control RNA was used for cDNA synthesis using Superscript reverse transcriptase (Life Technologies) and 18-mer oligo-dT primers. The cDNA derived from TCC or normal bladder material was further labelled with Cy3-dCTP or Cy5-dCTP (Amersham, UK), respectively. The labelled cDNA was used for hybridisation to the UniGEM 1 DNA chip, which contained approximately 10 000 human cDNA clones (IncyteGenomics, Palo Alto, CA, USA). Hybridisation, washing, and scanning of the slides were carried out as previously described (Schena *et al*, 1996).

Data from GEM experiments were analysed by using the GEMTools software package (Incyte). cDNA clones displaying differential values were sequenced and their identity was defined.

### MAGE-A8 expression by RT–PCR

TCC lines were grown in the tissue culture, and the cells were harvested for RNA isolation. Tumour samples were collected in the Carmel Medical Center (Haifa, Israel) by transurothral resection of the bladder tumour (TURBT), and immediately frozen in liquid nitrogen. Isolation of total RNA was performed by TRIREAGENT (Molecular Research Center, Cincinnati, OH, USA) according to the manufacturer's instructions. Reverse transcription was performed on 5 *μ*g of total RNA using an oligo (dT) 18-mer as a primer. The cDNA quality was assured by expression of GAPDH, a widely expressed metabolic gene. cDNA corresponding to 500 ng of total RNA was subjected to PCR amplification for 40 cycles, as follows: 1 min at 92°C; 1 min at 62°C for MAGE-A, 8 or 56°C for GAPDH; 1 min at 72°C. Amplification was followed by a 10 min incubation at 72°C. The following primers were employed: MAGE-A8 sense primer – 5′ ccc cag aga agc act gaa gaa g 3′; MAGE-A8 anti sense primer – 5′ ggt gag ctg ggt ccg gg 3′; GAPDH sense primer – 5′ ACC ACA GTC CAT GCC ATC AC 3′; GAPDH anti-sense primer – 5′ TCC ACC ACC CTG TTG CTG TA 3′. Products of the reaction were subjected to electrophoresis on 1.5% agarose gels and monitored under UV light.

### Peptide synthesis

Peptides were synthesised on an ABIMED AMS 422 multiple peptide synthesiser (Abimed, Langenfeld, Germany), employing the *α*-*N*-fluorenylmethoxy-cartonyl (Fmoc) strategy following the commercially available manufacturer's protocols. Peptide-chain assembly was conducted on a 2-chlorotrityl-chloride resin (Novabiochem, Laufelfingen, Switzerland). Crude peptides were purified to homogeneity by reversed-phase HPLC on a semipreparative silica C-8 column (250 × 32 × 10 mm^3^; Lichnonorb RP-8; Merck, Darmstadt, Germany). Elution was accomplished by a linear gradient established between 0.1% TFA in water and 0.1% TFA in 70% acetonitrile in water (v v^−1^). Composition of the products was determined by amino-acid analysis (automatic amino-acid analyzer; Dionex, Sunnyvale, CA, USA) after extraction acid hydrolysis. Molecular weight was ascertained by mass spectrometry (VG Tofspec; Laser Desorption Mass Spectrometry; Fisons, Manchester, UK). The MAGE-A8-encoded peptide sequences are presented in Table 3. Other peptides that served in this study are HLA-A2.1-binding peptides of HIV (TLNAWVKVV) and Tyrosinase (YMNGTMSQV).

### Peptide-binding assay and FACS staining

Peptide binding to MHC molecules was monitored by stabilisation assays, in which TAP-deficient, HHD-transfected RMA-S cells (RMA-S/HHD) are loaded with a peptide, and HHD expression on the cell surface is monitored by FACS. In more detail, RMA-S/HHD cells were washed with PBS and cultured overnight in serum-free optiMEM medium (Life Technologies, Paisley, UK), at 26^O^C, in order to increase the surface expression of the HHD molecule. In all, 1, 10, and 100 *μ*M of each peptide were added to 5 × 10^5^ RMA-S/HHD cells in 100 *μ*l volume of opti-MEM for 3 h at 26°C, and cultures were shifted to 37°C for 2 h, to decrease the background level expression of peptide-free HHD molecules. The cells were then stained with the HLA-specific W6/32 mAb (Serotek, UK) and monitored by FACS (Becton Dickinson, Canberra, Australia).

### Vaccination

HHD mice were immunised i.p., three times at 7-day intervals, with 2 × 10^6^ irradiated (5000 rad), peptide-loaded RMA-S/HHD/B7.1 transfectants. Alternatively, mice were immunised with 100 *μ*g of peptide in 100 *μ*l of Incomplete Freund's Adjuvant (IFA) (DIFCO, Detroit, MI, USA) injected twice to the tail-base, in 14 days interval. For immunisation with cholera toxin (CT), 1 *μ*g of CT (List Biological Laboratories, Inc, Campbell, CA, USA) was mixed with 12.5 *μ*g of the immunising peptide, in a volume of 16 *μ*l, and dropped into the nozzle of anaesthetised mice. Immunisation with CT was carried out three times, in 7-days intervals.

For all the vaccination modes, spleens were removed on day 10 after the last immunisation, and splenocytes were re-stimulated *in vitro*, with one-third of the cells pre-pulsed with 100 *μ*M synthetic peptides in optiMEM (Life Technologies, Paisley, UK) for 2 h at 37°C, 5% CO_2_. Re-stimulated lymphocytes were cultured in RPMI-HEPES medium containing 10% FCS, 2 mM glutamine, combined antibiotics, 1 mM sodium pyruvate, 25 mM HEPES, 5 × 10^−5^ M *β*ME, and 1% NEAA for 5 days. Viable cells (effectors) were separated by Lympholyte-M (Cedarlane, Hornby, Canada) gradients, re-suspended, and admixed at different ratios with 5000 ^35^S-methionine-labelled target cells. When served as target cells, EL4-HHD cells were pulsed with 100 *μ*M peptide in optiMEM at 37°C for 3 h. Cytolytic assays were carried out as described previously (Carmon *et al*, 2000), in four effector-to-target concentrations, from 100 : 1 to 12.5 : 1. In all the experiments, the spontaneous release was <25% of maximal release. Error was <5% of the mean of the triplicates. Percent of specific lysis was calculated as follows: % lysis=(c.p.m. in experimental well−c.p.m. spontaneous release)/(c.p.m. maximal release−c.p.m. spontaneous release) × 100.

### *In vitro* priming of human CTL

Leukapheresis products of two healthy donors were obtained from Barzilai Medical Center (Ashkelon, Israel) according to the Declaration of Helsinki Principles. Peripheral blood mononuclear cells (PBMC) were isolated by centrifugation on Ficoll-Plaque Plus gradients (Amersham, Sweden). The procedure was carried out according to Thurner *et al* (1999), with minor modifications. Briefly, *in vitro* priming was performed over autologous dendritic cells (DC) that were prepared from monocytes by IL-4 and GM-CSF treatment and matured with an IL-1*β*, IL-6, TNF-*α*, and PGE_2_ cocktail. Dendritic cells were pulsed with synthetic peptides and PBMC were supplemented with IL-7. After 2 days, IL-2 was added and renewed every 3 days. Second and third stimulations were carried out on days 7 and 14 on peptide-pulsed, irradiated monocytes. A fourth stimulation was carried out with autologous DC, as above. At 7 days after the fourth stimulation, lymphocytes were harvested and cytolytic assays were performed. In all the experiments, the spontaneous release was <25% of maximal release. The error was <5% of the mean of the triplicates. The percentage of specific lysis was calculated as above.

## RESULTS

We performed a cDNA chip-based differential display analysis, in order to identify overexpressed genes of bladder carcinoma, as compared to normal bladder mucosa. A pool of 10 normal and 10 tumour samples was hybridised to a cDNA chip containing 10 000 cDNA sequences. The cDNA fragments are of 500–5000 bases, originating from EST clones, and 40% are identified as known genes. Hybridisation sensitivity is up to one part in 100 000, which is sufficient to detect single copy expressed genes. Two-fold changes of expression of any gene on the microarray are detectable.

About 480 genes, out of 10 000 of the DNA chip, were found to be induced two-fold or more in the TCC tissue, compared to normal bladder tissue. Among the overexpressed genes of the tumour is the gene for the MAGE-A8, which was amplified by 7.8-fold, with background level of expression in the normal tissue (Table 1

**Table 1:** The 20 most amplified genes of the TCC samples, as compared to normal bladder samples

**Gene**	**Accession number**	**Fold of amplification**
*Homo sapiens* keratin 6 isoform K6d (KRT6D) gene	AA036948	53.3
ESTs	W74581	52.3
ESTs	R36967	38.9
Human nonspecific crossreacting antigen mRNA	AA054073	36.5
KERATIN	AA026418	23.9
GLYPICAN PRECURSOR	W725759	23.2
ESTs {347362}	W81087	14.1
INTERLEUKIN-8 PRECURSOR	Y00787	13.4
ESTs	H93048	11.6
KERATIN	AA031736	11.3
C-PROTEIN	N66106	10.9
ESTs	W77796	10.5
KERATIN	AA026642	10.4
MUCIN 1 PRECURSOR	N27731	9.1
CORNIFIN B	W72751	9
PLASMINOGEN ACTIVATOR INHIBITOR-2	AA032091	8.3
Human MAGE-8 antigen (MAGE8) gene	U10693	7.8
ESTs	H81053	7.6
CYSTATIN A	AA022874	7.5
OSTEOPONTIN PRECURSOR	AA021512	7.5

For technical details, see Materials and methods section.

). Since many MAGE family genes encode for TAA, we chose to further examine the MAGE-A8 as a TAA.

**Figure 1:**
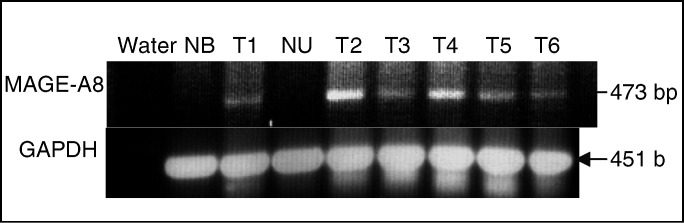
MAGE-A8-gene expression in fresh tissue samples. Fresh TCC samples (T1–T6), normal bladder (NB), and normal ureter (NU) were collected and immediately frozen in liquid nitrogen. RNA was isolated, cDNA was reverse-transcribed and served as a template for PCR amplification with MAGE-A8-specific primers. GAPDH-specific primers served to assure the cDNA quality. A DNA-free sample served as a negative control for the reaction.

To assess and validate the MAGE-A8 gene expression in TCC, we examined its expression in fresh tumour samples and TCC lines by RT–PCR. We examined 23 tumour samples, all collected by transurothral resection of the bladder tumour (TURBT), and immediately frozen in liquid nitrogen. RNA was isolated, and reverse transcribed into cDNA. The cDNA quality of all the examined samples was assured by GAPDH expression. Of the 23 examined samples, 17 were found to express the MAGE-A8 gene (74%) and no expression was detected in normal bladder and normal ureter samples (Figure 1

). The PCR primers are designed to bind to the second and third exons in the MAGE-A8 gene, to avoid false-positive results due to genomic DNA contamination. We also examined the MAGE-A8 gene expression in human TCC lines (Table 2

**Table 2:** MAGE-A8 gene expression in TCC lines

**Cell line**	**Tumour grade**	**MAGE 8 expression**
T24	III	+
UM-UC3	Unknown	+
RT4	I – papillary	−
J82	III	−
TCCSUP	IV – metastases	−
5637	II	+
1196	IV	−
SW780	I	+

RNA was extracted from eight TCC lines, cDNA was reverse transcribed and served as a template for PCR amplification with MAGE-A8 specific primers. In all the examined samples, the cDNA quality was assured by GAPDH expression.

). Out of eight examined, four were found to express the MAGE-A8 gene, among them the T24 cell line.

The MAGE-A8 protein sequence was screened for HLA-A2.1-binding motifs. This analysis was performed through a world-wide web interface (http://www-bimas.dcrt.nih.gov/ molbio/hla_bind/index.html). This software scores every possible peptide along the sequence for MHC binding. Every amino acid in the evaluated peptide is scored according to its relative contribution to the binding (Parker *et al*, 1994). We synthesised six peptides that were predicted the highest for HLA-A2.1 binding, for further study (Table 3

**Table 3:** MAGE-A8 peptides

**Peptide**	**Position**	**Sequence**
8.1	24–32	GLMDVQIPT
8.2	111–119	ALDEKVAEL
8.3	115–123	KVAELVRFL
8.4	45–53	LIMGTLEEV
8.5	179–187	YILVTCLGL
8.6	161–169	MQVIFGIDV

**Figure 2:**
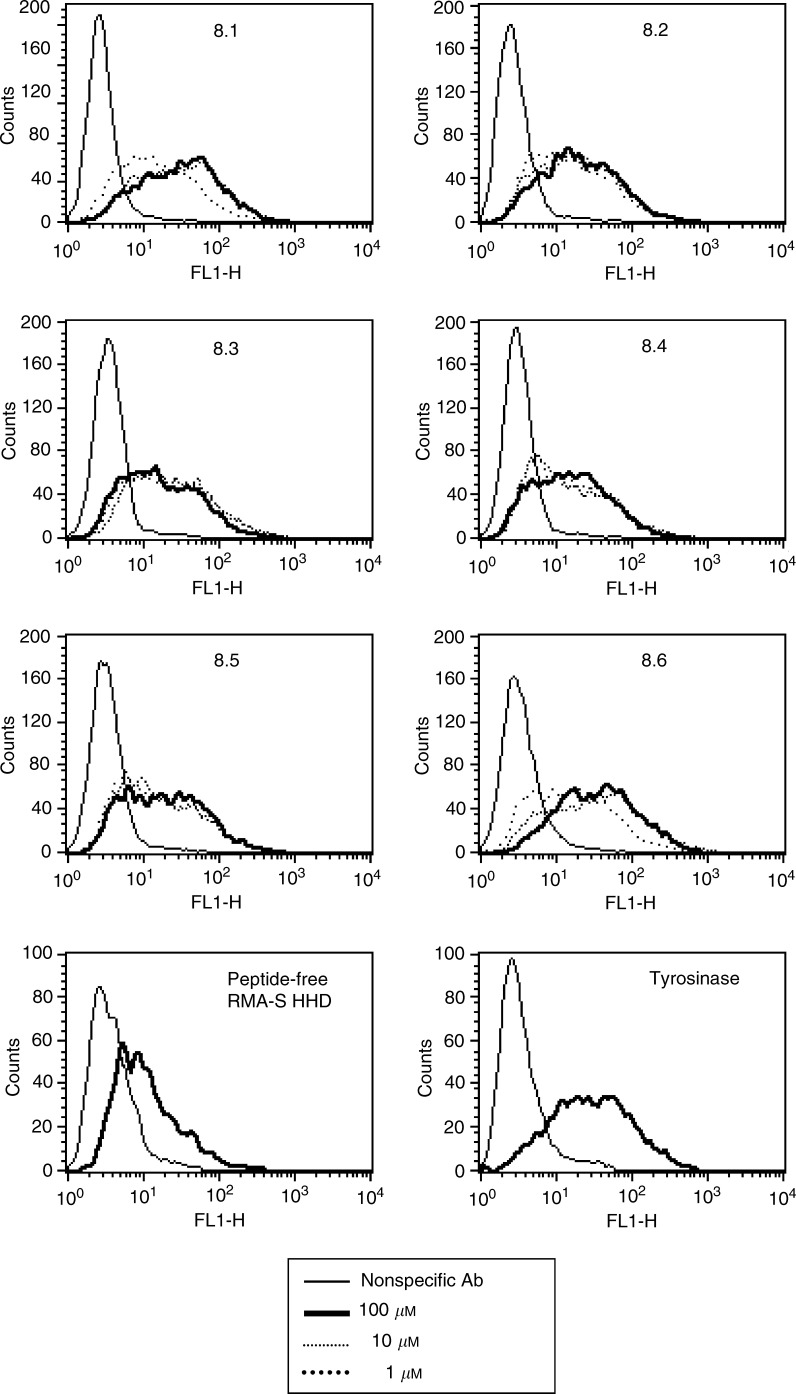
MAGE-A8-encoded peptide binding to HLA-A2.1. Peptides were loaded on the TAP-2-defficient, RMA-S HHD cells, at three different concentrations, for 3 h at 26°C, and shifted for two more hours to 37°C (see Materials and methods). Cells were then washed, stained with the BB7.2 monoclonal antibody (specific for HLA-A2) or with the corresponding isotype control (not shown), and monitored by FACS. The tyrosinase-encoded peptide, a high binder of HLA-A2.1, served as a positive control, and a background level of peptide-free cells is demonstrated. Representative experiment out of three.

The position along the protein sequence and single-letter amino-acid sequence are presented.

). To confirm the MAGE-A8 peptides binding to HLA-A2.1, we examined their potential to stabilise empty HLA molecules. TAP-2-deficient, HHD-transfected, RMA-S cells (RMA-S/HHD) were loaded with individual peptides at three different concentrations (100, 10, and 1 *μ*M), and surface expression of HLA-A2.1 molecules was monitored by FACS (Figure 2

). All the six MAGE-A8 peptides were strong binders of HLA-A2.1.

**Figure 3:**
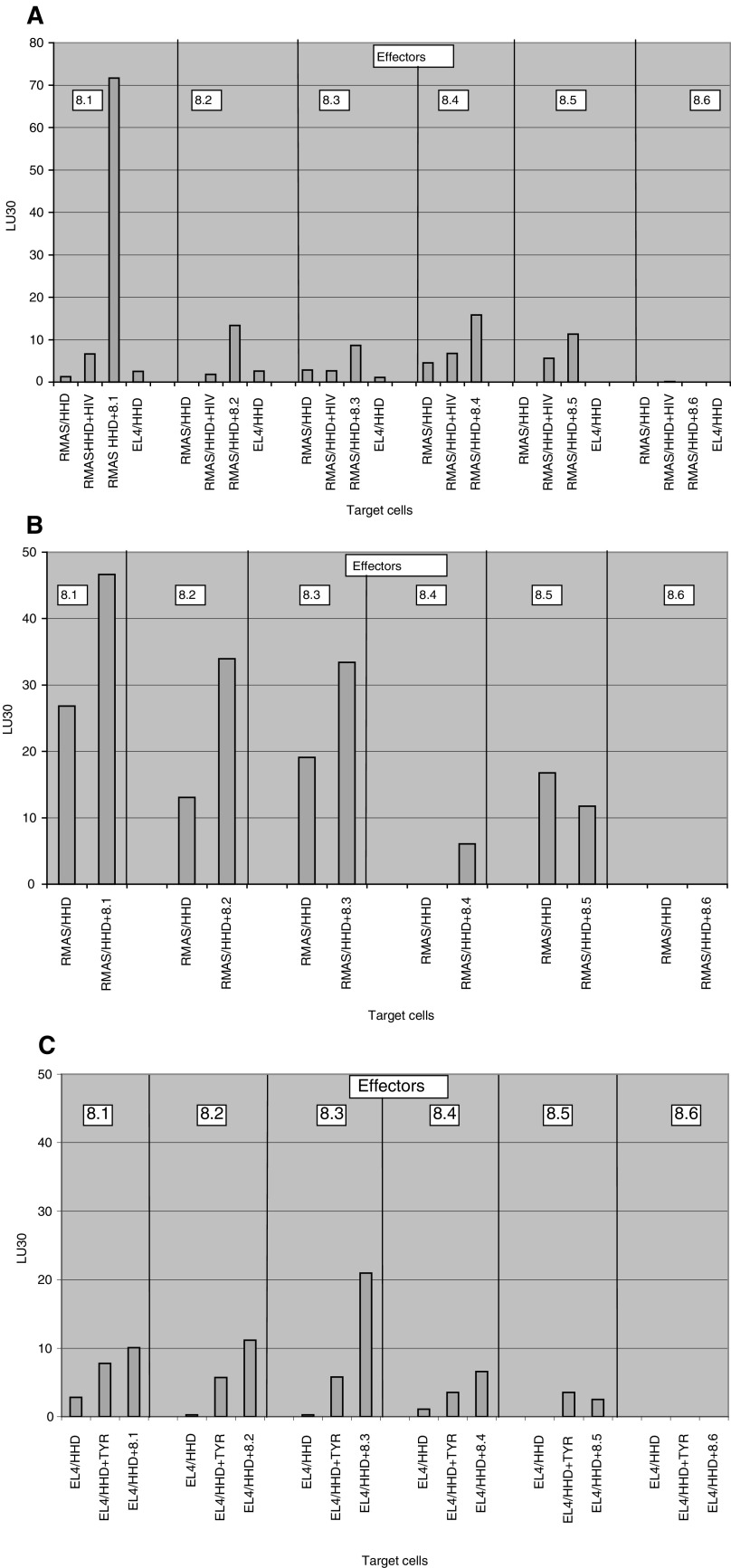
Immunogenicity of the MAGE-A8 peptides determined by three modes of vaccination. (**A**) HHD mice were immunised intranasally, three times, with 12.5 *μ*g of individual peptides, dissolved in PBS and supplemented with 1 *μ*g of CT. At 10 days following the third immunisation, splenocytes were re-sensitised with the immunising peptide for 4 days, and cytotoxicity was determined as described in Materials and methods. (**B**) Immunisation of HHD mice was performed with 100 *μ*g of individual peptides, in IFA, injected twice in 14 days interval into the base of the tail. Cytotoxicity was determined subsequent to splenocyte re-sensitisation. (**C**) HHD mice were vaccinated three times, in 7 days intervals, with RMAS/HHD/B7 cells, loaded with individual peptides. Splenocytes were re-sensitised with the immunising peptide and cytotoxicity was monitored using peptide-pulsed EL4-HHD as targets. Data are presented as lytic units 30 (LU30), which are defined as the number of cells required for 30% target lysis (see Materials and methods for more details). Representative experiment out of three.

Screening for the immunogenicity of HLA-A2.1-binding peptides was carried out in HHD mice, which are eliminated for the murine class I MHC, and transgenic for HLA-A2.1/D^b^-*β*2-microglobulin single chain (see Materials and methods, and Pascolo *et al*, 1997). The *α*3 region of the HHD molecule is of murine origin, to enable efficient interaction with CD8 molecules. The MAGE-A8 peptide immunogenicity was examined in three modes of vaccination. HHD mice were immunised with individual peptides, splenocytes were re-stimulated *in vitro* with the immunising peptide, and cytotoxicity against cells loaded with the immunising peptide was measured. Immunisation with CT was carried out intranasally. Peptide 8.1 is immunogenic, and peptides 8.2, 8.3, 8.4, and 8.5 are of low immunogenicity, and no immunogenicity was detected for peptide 8.6 (Figure 3A

). The second mode of administration was with individual peptides in IFA, injected to the base of the tail (Figure 3B). Peptides 8.1, 8.2, 8.3, and 8.4 showed higher lysis of peptide-loaded RMA-S/HHD cells, than of unloaded RMA-S/HHD or RMA-S/HHD loaded with an irrelevant HLA A2-binding peptide derived from HIV (not shown). CTL induced by peptides 8.5 or 8.6 do not show any specific lysis. The third mode of immunisation was with irradiated RMS-S/HHD/B7 cells, loaded with individual peptides and injected into the peritoneal cavity (Figure 3C). The highest immunogenicity was observed for peptide 8.3,while peptide 8.2 shows low specific lysis. For vaccination with peptide-loaded RMA-S/HHD/B7 cells, peptide-pulsed EL4-HHD served as target cells, to avoid cross-reaction with RMA-S/HHD/B7 target cells.

In the light of these data, we decided to further analyse the antigenicity of peptides 8.1 and 8.3.

**Figure 4:**
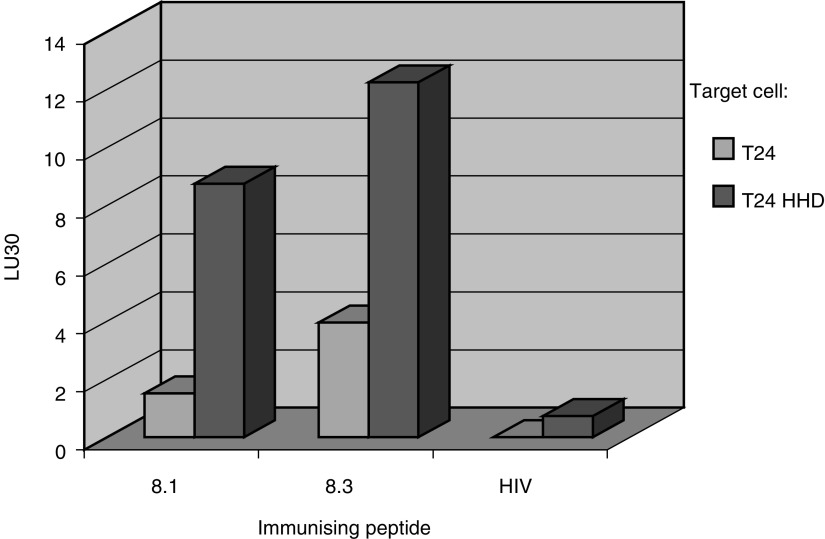
Antigenicity of peptides 8.1 and 8.3. HHD mice were vaccinated three times, in 7 days intervals, with RMA-S/HHD/B7 cells, loaded with peptides 8.1, 8.3, or an HIV-encoded peptide. At 10 days following the third immunisation, spleens were removed and splenocytes were re-sensitised with the immunising peptide, for 4 days. Cytotoxicity was determined against the T24 or T24-HHD cells, both expressing the MAGE-A8 gene. Data are presented as lytic units 30 (LU30), which is defined as the number of cells required for 30% target lysis (see Materials and methods for more details). Representative experiment out of three.

Peptides 8.1 and 8.3 were found to be the most immunogenic in more than one of three modes of vaccinations, that is, both peptides can elicit an efficient immune response towards the same peptide-pulsed target cells. However, the existence of these peptides as natural epitopes on tumour cells should be demonstrated. Therefore, we immunised HHD mice with each of the peptides loaded on RMA-S/HHD/B7 cells and the cytotoxic activity against the T24-HHD cells was calculated. T24-HHD is a human TCC line, expressing the MAGE-A8 gene (Table 3), which was transfected with the HHD construct (data not shown). Both peptides 8.1 and 8.3 elicited an HLA-A2.1-restricted specific response to the T24-HHD cells (Figure 4

), such a response was not observed when HHD mice were immunised with the HIV-derived unrelated peptide. These results show that both the 8.1 and 8.3 peptides are processed and presented on HLA molecules of the T24-HHD tumour cells.

**Figure 5:**
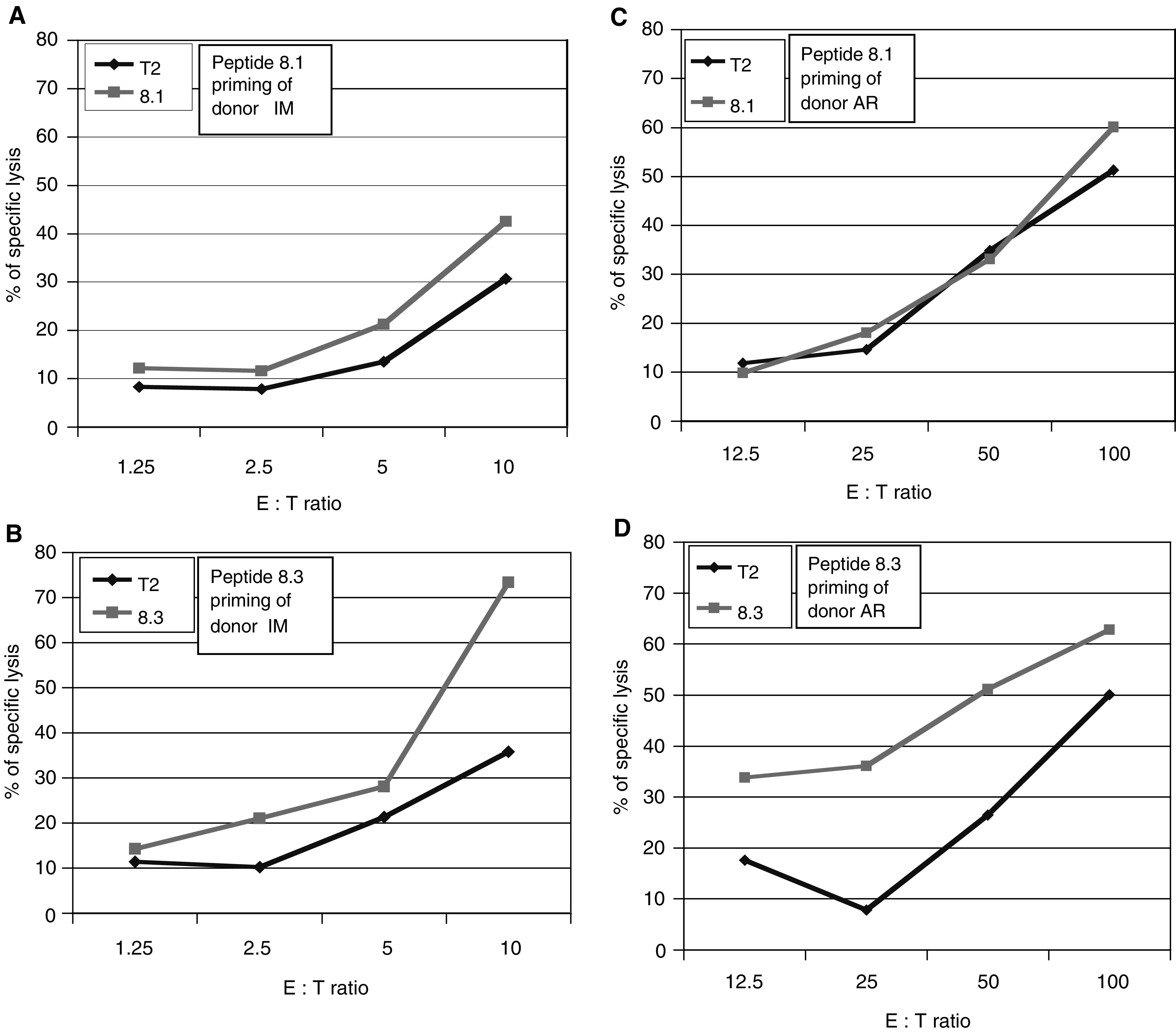
*In vitro* priming of a CTL response in human lymphocytes towards peptides 8.1 and 8.3. Peripheral blood mononuclear cells were collected by leukapheresis from two healthy male donors, designated IM (**A**, **B**) and AR (**C**, **D**). Lymphocytes were primed with peptide-pulsed autologous DC. Peripheral blood mononuclear cells were supplemented with IL-7, and, 2 days later, IL-2 was added and renewed every 3 days. The next two stimulations were carried out every 7 days over peptide-pulsed monocytes, and a fourth stimulation was carried out with peptide-pulsed autologous DC. At 7 days following the last stimulation, cytotoxicity was determined against peptide-pulsed or nonpulsed T2 cells as targets.

HHD mice can predict well peptide immunogenicity, yet immunogenicity has to be examined also in humans. To examine whether peptides 8.1 and 8.3 can prime CTL in human lymphocytes *in vitro*, PBMC of healthy donors were collected by leukapheresis, and kept as frozen aliquots. Lymphocytes were stimulated in the presence of IL-2 and IL-7 four times. The first and fourth stimulations were carried out with DC loaded with peptide 8.1 or 8.3. The second and third stimulations were accomplished by peptide-pulsed irradiated monocytes. At 7 days following the fourth stimulation, cytotoxicity assays were performed. For the donor IM, peptides 8.1 and 8.3 primed a specific cytotoxic response at low E : T ratios (Figure 5A, B

). For AR, peptide 8.3, but not 8.1, primed a specific response (Figure 5C, D). Therefore, the immunogenic potential of peptide 8.3 was demonstrated in both donors, and that of peptide 8.1 in one donor. Hence, there is no absolute ‘tolerance barrier’ that prevents priming of human cytotoxic response towards the MAGE-A8 peptides.

## DISCUSSION

This study is aimed at identification of TAA peptides of human TCC. The first human TAAs were identified by genetic methods, utilising T cells against autologous cancer cells (van der Bruggen *et al*, 1991; Kawakami *et al*, 1994). Alternatively, TAA peptides were identified by biochemical methods (Cox *et al*, 1994; Mandelboim *et al*, 1994) or peptide libraries (Chen, 1999). Our study is based on a different strategy, of ‘reverse immunology’. Most methods depend on patient-derived T cells, which already failed to react against the tumour. Reverse immunology aims to identify candidate antigens, and to look for possible epitopes within them.

To identify potential tumour antigens, gene expression profiles of TCC *vs* normal bladder were examined by DNA microarray. The analysis provided us a comprehensive source of information, and enabled to identify expression of genes that were not known to correlate with TCC.

Several parameters are required for a probable TAA gene. First, a high expression level is required, in order to yield higher epitope expression level on the cell surface (Yewdell, 2001). A second requirement is for minimal expression level in the normal tissue, to avoid autoimmune complication. For a higher clinical relevance, TAA genes should be expressed in a high percentage of tumours, preferentially of various types. The MAGE-A8 fits these requirements, it was amplified by 7.8-fold, with no evidence of expression in the normal tissue. RT–PCR analysis revealed high expression level in fresh tumour samples (17 of 23), no expression in samples of normal bladder and ureter (Figure 1), and four of eight TCC lines (Table 2). As was previously described, 46% of hepatocellular carcinomas (Tahara *et al*, 1999) and 44% of colorectal carcinomas (Hasegawa *et al*, 1998) expressed the MAGE-A8 gene. Moreover, expression of MAGE-A8 gene in adult tissue is limited to the male germ line, which does not express HLA molecules, and to the placenta (De Plaen *et al*, 1994).

Ideally, a TAA would be functionally related to the tumorigenesis and to metastases formation process. Since the role of MAGE-A8 is not defined, its involvement in tumour formation and progression is not clear. MAGE genes are expressed as a result of DNA demethylation in tumour cells (Barker and Salehi, 2002). Therefore, it is not clear whether MAGE genes expression is part of the tumour formation process, or its byproduct. It was recently shown that MAGE genes are expressed early in lung carcinogenesis (Jang *et al*, 2001), and therefore may serve both for early detection and for treatment of cancer cells. Clinical trials with MAGE peptides are currently carried out, mainly for melanoma (Parmiani *et al*, 2002). Melanoma regression following vaccination with MAGE-3 peptide was demonstrated, with low-frequency CTL response (Coulie *et al*, 2001). Another study (Nishiyama *et al*, 2001) describes the use of autologous DC pulsed with an HLA-A24-restricted MAGE-A3-encoded peptide. In three of four patients, significant reduction in the size of metastases was observed, and a complete response was detected in one patient. This pilot study reveals the potential use of TAA peptides for TCC immunotherapy.

We next looked for potential T-cell epitopes of MAGE-A8, utilising the HHD system. It was shown that HHD mice selected the same immunodominant CTL epitopes as recognised by PBL in influenza-infected HLA-A2.1 individuals (Pascolo *et al*, 1997). Unlike traditional HLA transgenic mice, HHD mice T-cell response is HLA-A2.1 restricted, with no murine background. It was shown that the TCR repertoire of HHD mice in response to HLA-A2.1-restricted antigens is larger that that of traditional HLA-A2.1 transgenic mice (Firat *et al*, 2002). Thus, these mice are presumably an ideal tool for the identification and characterisation of potential tumour-derived HLA-A2.1-restricted CTL epitopes.

A potent TAA peptide epitope should be a good HLA binder, immunogenic, and antigenic. To this end, we identified six HLA-A2.1-binding peptides of MAGE-A8 (Table 3, Figure 2). The immungenicity of peptide epitopes may vary between different modes of vaccination (Firat *et al*, 1999). The immunogenic potential of the MAGE-A8 peptides was examined in three vaccination modes; peptides delivered intranasally with CT as a mucosal adjuvant, peptides in complete Freund's adjuvant (CFA) injected to the tail base, and peptide loaded on RMA-S/HHD/B7 cells injected intraparitoneally (Figure 3A–C, respectively). All these modes of vaccination were proven to give efficient immunisation in mice (Porgador *et al*, 1997; Carmon *et al*, 2002; Firat *et al*, 2002). Taking into account that peptides 8.1 and 8.3 were immunogenic in more than one mode of vaccination, we decided to further investigate the potential of peptides 8.1 and 8.3 to serve as TAA epitopes.

The screening system enables us to identify immunogenic peptides, but antigenicity is not addressed. The potential of synthetic peptide to induce an immune response does not tell about its existence as a natural epitope. To this end, we examined whether T cells specific for each of the peptides can specifically lyse the T24-HHD cells, as compared to T24 cells (Figure 4). T24 cells express the MAGE-A8 gene (Table 2), and so do T24-HHD cells (not shown). Both peptides 8.1 and 8.3, but not the irrelevant HIV peptide, induced a cytotoxic response against the T24-HHD cells. The cytotoxic response is HLA-A2.1 restricted, since lysis of T24 cells was at a low level. Thus, both peptides 8.1 and 8.3 are presented by HLA-A2.1 molecules on the surface of MAGE-A8-expressing cells.

The immunogenic potential of the 8.1 and 8.3 peptides was demonstrated in HHD mice. Since tolerance to immune-dominant peptides from self proteins was demonstrated (Theobald *et al*, 1997; Colella *et al*, 2000), we tested whether these peptides can prime CTL in humans. For peptide 8.1, immunogenicity was shown in one of two healthy donors, and for peptide 8.3 in both donors (Figure 5A–D). The killing activity was limited, as compared to the background level of nonpulsed cells, but lack of activation was detected for peptide 8.1 with AR donor only. Moreover, in the case of IM, cytotoxicity was observed in a relatively low effector-to-target ratio. We therefore conclude that both peptides are capable of priming cytotoxic activity in human lymphocytes. CD8^+^ cells in HHD mice are selected on a single allele of HLA-A2.1. Therefore, negative selection is limited, and the TCR repertoire is wider than in human (Firat *et al*, 2002). TCR selection in human is carried out through up to six HLA alleles for class I HLA, and that could limit the TCR repertoire. Even though, priming of cytotoxic cells is within reach.

The search for TCC-associated antigens is limited. There are only few cases in which TCC-specific TAA epitopes were identified (Gueguen *et al*, 1998; Heidecker *et al*, 2000), in spite of the immunogenic potential of this tumour, since TCC is among few types of cancers that are immunotherapy responsive. Intravesical delivery of BCG is a standard therapy for superficial bladder cancer, and has positive outcomes on tumour recurrence rate, disease progression, and prolongation of survival (Nseyo and Lamm, 1997). A positive clinical outcome was observed by other modalities of immunotherapy, such as the use of *α*-interferon (*α*IFN) and keyhole limpet hemocyanin (KLH). The use of TAA peptides is advantageous, since it can target the tumour cells specifically, with lower side effects than nonspecific immunotherapy. A combined therapy of local immune stimulation with the use of specific tumour antigens could be of great clinical value. The MAGE-A8 peptides, as well as other TAA peptides, could serve for such a therapy.
